# To What Extent May the Female Genitals Be Saved from Mutilation?1Read before Section on Surgery, Buffalo Academy of Medicine, December 2, 1902.

**Published:** 1903-01

**Authors:** J. Henry Dowd

**Affiliations:** Buffalo, N.Y., 378 Franklin Street.


### To What Extent May the Female Genitals be Saved From Mutilation?[1]

By J. HENRY DOWD, M. D., Buffalo, N. Y.

WHEN one considers the vast amount of suffering among our female population, practically all such giving the history of trouble following marriage, and we as medical men knowing that practically four-fifths of it is due to preventable causes, must not the question arise, what can be done to prevent it?

German authorities tell us that at least 85 per cent. of inflammatory disease of the female genitals is due to gonorrhea, fully 40 to 45 per cent. of the operations on these organs being due to the same cause.

We may go a little further, taking statistics which are undoubtedly true and we find that about 30 per cent. of sterile marriages, and no less than 10 to 20 per cent. of blindness, before the introduction of Credé's method of treatment, due to the same cause.

Neisser places gonorrhea next to measles in the order of contagious diseases, and furthermore claims that in some European cities no less than three-fourths of the population have had the disease. It can scarcely be said that the disease is as prevalent in America, yet it may be safely stated that at least 4 out of 10 of the male population have been its victims. Some authorities claim that gonorrhea is never cured, that its complications which may arise are never fully eradicated. This the writer must dispute and affirms that gonorrhea, if treated rationally, is curable; that is, practically all cases; complications which have arisen can be permanently eradicated, the patient put in a condition where there is no danger of infection either from gonococci, strepto- or staphylococci, the two latter being seen in the shreds and pus found in the urine long after there is no discharge and the former cocci have disappeared.

Three of the most prolific causes of inflammation are the strepto-, staphylo-, and gonoccoci, the first two being usually mild in the results produced as compared with the latter. Granting the above to be a fact, it also must be admitted that if the microscope shows the gonococci to be absent, but strepto- or staphylococci are present, infection is possible. If the case pro-

1. Read before Section on Surgery, Buffalo Academy of Medicine, December 2, 1902.

gresses favorably, about the sixth or eight week there is an absence of discharge, but if the urine is examined it will be found to contain floating débris, any or all of which the microscope will show to be composed of pus, epithelium, mucus, normal urethral bacteria, strepto- and staphylococci, but usually no gonococci. This débris is due to different causes, chief of which, especially in the early stage, is the fact that inflammation is still present, but not of sufficient intensity whereby pus will be produced in quantity sufficient to appear at the meatus. One other factor in causing its expulsion from the canal is coitus. The longer the interval, the more secretion; therefore we find the greatest amount in the morning, the canal not having been flushed for seven or eight hours. It must be evident that when the canal is free, *i. e.*, in an apparently normal condition, if infecting material is contained in the numerous follicles of the prostate, ejaculatory ducts, vesicles, Cowper's gland, and the like, it is expelled at this time. When the latter condition exists there may be an entire absence of any and all objective symptoms, but by careful questioning subjective symptoms may be elicited that will point to a condition in these parts showing that they are not normally functuating.

A word as to infection of the female genitals by gonorrheal or other forms of inflammation. It is a well known fact that all women have more or less discharge, commonly called leucorrhea, even virgins having this at times to an extent necessitating wearing of a protective; and, further, intense inflammation may exist around the cervix, with no other symptoms presenting. Practically the same condition may be present of a gonorrheal nature and with no other symptoms than already mentioned presenting. That gonorrhea may exist in the female unknown, is easily accounted for by the fact, that it is rarely that intercourse is attempted by the male until there is no discharge at the meatus or only a very slight secretion, brought forward by firm stripping of the organ. In this condition the infecting material when expelled, is deposited not at the vulva, where the parts may become inflamed accompanied by ardor urinæ, swelling and a sense of uneasiness, but against the cervix, a practically insensitive part of the anatomy. Accepting as a fair estimate that about 40 per cent. of males have had gonorrhea, that not 25 per cent. of these are permanently cured, but have infecting cocci, not necessarily gonococci, in the urethra, is it little wonder, as heretofore stated, that about 85 per cent. of inflammatory disease of the female organs can be traced to, and give a history of trouble originating after marriage?

In speaking of a remedy for this condition contention will not be made that the 85 per cent. of post-marriage inflammations of the female genitalia can be prevented, but the medical man who will remove all sources of infection from the patient before pronouncing him cured will have the satisfaction of knowing if his patient ever marries, that the genital organs of one female will remain intact.

A male patient having had inflammation of the genital tract may be considered well and in an aseptic condition when there is no discharge from the meatus containing bacteria or pus cells, nor can it be reëstablished by liquor or coitus, with no symptoms referrable to the testicles, prostate or seminal vesicles, the urine being clear of all pathological débris. One and the chief cause why so many men are circulating among the community having infecting bacteria in their urethræ, is the fact that the original trouble was treated upon a lump, or contract basis, instead of by the visit.

Inflammation of the urethra extending over eight weeks is always accompanied by involvement of the deeper parts, this necessitating at times long, careful and scientific treatment to bring about a normal condition. When the number of visits are counted, the wear and tear on the medical man's nervous system taken into consideration, the case extending into months before what may be considered a cure is obtained, is it any wonder, in a cure-you-for-ten-dollars case, the patient is discharged the moment, perchance, by a strong injection or the passage of a sound, pus ceases to flow?

*Conclusions.*—Gonorrhea in the male being curable in at least 95 to 98 per cent. of cases, and I mean by that placed beyond possible danger of conveying infection, it is only natural to make the bold statement, if the disease is healed rationally and until all danger of infection has been obliterated, the percentages of inflammation in the female genitals should be reduced to a minimum.

The argument that the druggist treats three out of five of these cases has no standing, for the reason, that they do not stop the discharge in one out of ten, the remaining nine having sooner or later to consult a physician.

378 FRANKLIN STREET.

